# 2792. Determining the activity of imipenem/relebactam (I/R) plus aztreonam (ATM) against constitutive Pseudomonas derived cephalosporinase (PDC)- and metallo-β-lactamase (MBL)-producing *Pseudomonas aeruginosa* in a hollow fiber infection model (HFIM)

**DOI:** 10.1093/ofid/ofad500.2403

**Published:** 2023-11-27

**Authors:** J Nicholas O’Donnell, Kelly E Moolick, Avery I Nahorniak, Maxwell J Gifford, Katherine Young, Thomas Lodise

**Affiliations:** Albany College of Pharmacy and Health Sciences, Albany, New York; Albany College of Pharmacy and Health Sciences, Albany, New York; ACPHS, Albany, New York; Albany College of Pharmacy and Health Sciences, Albany, New York; Merck, Rahway, New Jersey; Albany College of Pharmacy and Health Sciences, Albany, New York

## Abstract

**Background:**

Few treatment options exist for serious infections caused by MBL-PSA. While ATM retains activity against some MBL-PSA, isolates often constitutively produce PDCs, resulting in resistance. I/R is a new β-lactam/β-lactamase inhibitor with expanded activity against constitutive PDC-producing PSA, but has no standalone activity against MBL-PSA. This study aimed to evaluate the effect of I/R plus ATM against PDC- and MBL-producing PSA in the HFIM.

**Figure:**
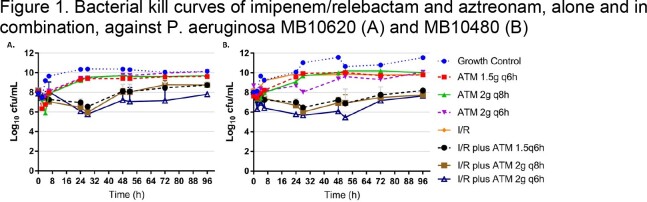

**Methods:**

Two isogenic, constitutive PDC-and MBL-producing PSA isolates (MB10480: IMP-1; MB10620: VIM-1) were studied at a starting inoculum of 8 log_10_ colony forming units (CFU)/mL in the HFIM over 96 hrs. Humanized exposures of I/R (500/250mg q6h as a 0.5 hr infusion) were evaluated alone and in combination with 3 ATM regimens (1.5g q6h; 2g q8h; 2g q6h as 2 hr infusions). Combination regimens were tested in triplicate with inoculum (log_10_ cfu/mL) averaged across runs. MIC testing with ATM and relebactam (set at 4 mg/L) was conducted on isolates recovered from the model at 96 hrs.

**Results:**

Monotherapy arms mirrored the growth control for MB10620 (ATM/relebactam MIC: 4/4 mg/L); combination arms resulted in bacteriostasis through 96 hrs. Maximal killing of each regimen (∼2 log_10_ CFU/mL; all units log_10_ cfu/mL change vs. baseline) was achieved through 32 hrs, followed by regrowth across all regimens (Figure 1). Bacterial counts at 96 hrs were similar to the starting inoculum for all combinations, but lowest for I/R plus ATM 2g q6h (I/R plus ATM 1.5g q6h: +1.75 CFU/mL; 2g q8h: +1.74 CFU/mL; 2g q6h: -0.20 CFU/mL;). Similar results were observed with MB10480 (ATM/relebactam MIC: 8/4 mg/L) (Figure 2). Maximal killing was achieved through 56 hrs (∼2 log_10_ CFU/mL), followed by regrowth across all combination regimens (I/R plus ATM 1.5g q6h: +0.19 CFU/mL; 2g q8h: -0.34 CFU/mL; 2g q6h: -0.35 cfu/mL). Across combination regimens for both isolates, 2 morphologies of PSA were recovered. Retested isolate MICs were occasionally, but not consistently, elevated by >2 dilutions (MB10620: 10/18; MB10480: 4/18).

**Conclusion:**

I/R plus ATM resulted in initial killing followed by regrowth over 96 hrs for constitutive PDC- and MBL-producing PSA isolates. Further study is needed to evaluate optimal dosing and suppression of regrowth in the HFIM.

**Disclosures:**

**J Nicholas O'Donnell, Pharm.D.**, Merck and Co, Inc.: Grant/Research Support **Thomas Lodise, Jr., Pharm.D., PhD**, merck: Grant/Research Support|merck: Honoraria

